# Circulating tumor cells and circulating tumor microemboli: role in venous thromboembolism in oncologic patients

**DOI:** 10.1042/BSR20253915

**Published:** 2026-01-09

**Authors:** Anna Paula Carreta Ruano, Jacqueline Aparecida Torres, Ludmilla Thomé Domingos Chinen

**Affiliations:** 1 Hcor, São Paulo, Paraíso, São Paulo-SP, Brazil (Rua Desembargador Eliseu Guilherme 147), Brazil; 2 Department of Farmacology, Federal University of São Paulo (UNIFESP), R. Três de Maio, 100 - Vila Clementino São Paulo, 04044-020, Brazil; 3 Fundação Amaral Carvalho, Jaú, São Paulo, (Av. Antônio de Almeida Pacheco, 1111-2 Zona Industrial; CEP: 17.213-700)

**Keywords:** biomarkers, cancer, circulating tumor cells, circulating tumor microemboli, liquid biopsy, thrombosis, venous thromboembolism

## Abstract

Venous thromboembolism (VTE) is one of the most frequent and serious complications in cancer patients, contributing significantly to morbidity, mortality, and increased healthcare burden. Circulating tumor cells (CTCs) and circulating tumor microemboli (CTMs), components of the liquid biopsy, have emerged not only as biomarkers of disease progression and therapeutic resistance but also as potential contributors to prothrombotic states in oncologic patients. In this review, we explore the biological and clinical relationships between CTCs/CTMs and the development of VTE, highlighting mechanistic insights involving tumor–platelet interactions, immune evasion, and endothelial dysfunction. We also discuss recent findings on the prognostic value of CTCs and CTMs for thromboembolic risk stratification, as well as technological advances in their detection. Understanding the role of these circulating tumor-derived elements may open new perspectives for personalized prevention and management of thromboembolic events in cancer patients.

## Introduction

An important characteristic of cancer is its ability to promote a state of hypercoagulation that can be observed through adverse clinical events. In the 19th century, Armand Trousseau described thrombophlebitis as an important event related to cancer by observing previously unknown visceral tumors in patient autopsies [1].

Over the years, Trousseau’s syndrome or cancer-associated thrombosis (CAT) has been widely studied. CAT is a set of diseases that can affect various venous systems throughout the body, being an aggravating factor for survival and for the increase of comorbidities among cancer patients [2]

The most common thrombotic event associated with cancer is venous thromboembolism (VTE), which includes deep vein thrombosis (DVT) and pulmonary embolism (PE) [3]. The incidence of VTE is substantially elevated in cancer patients, negatively impacting quality of life, treatment course, and, crucially, survival [4]. In fact, VTE ranks as the second leading cause of death in these patients, surpassed only by the progression of the neoplastic disease itself [5]. Understanding the underlying mechanisms of this complex interaction is fundamental for the development of effective management, prevention, and diagnostic strategies.

Studies show that the risk of developing VTE is 4.1 times higher in cancer patients compared with the general population. It is estimated that 4–20% of cancer patients develop VTE in the first months after diagnosis, with a cumulative incidence of 5% in the first six months. During chemotherapy, this risk reaches 6.5 times [6,7]. Thus, cancer therapy is considered intimately related to thromboembolism, which in turn constitutes the most important cardiovascular complication in patients receiving treatment [8,9].

In recent decades, scientific research has delved deeper into the factors that contribute to the hypercoagulable state associated with cancer. It is known that this is a multifactorial process, involving intrinsic tumor characteristics, such as histological type, stage, and aggressiveness; patient-related factors, as age and comorbidities; and extrinsic factors, such as oncological treatments (surgery, radiotherapy, and different types of chemotherapy) and patient mobility [10–14].

Based on these factors, the Khorana Score [15] was created, subsequently validated, and updated [16], a predictive model used to estimate the risk of VTE in ambulatory cancer patients who will start chemotherapy. It is based on 5 clinical and laboratory variables evaluated before treatment: tumor type, platelet count, hemoglobin (or use of erythropoiesis-stimulating agents), leukocyte count, and body mass index (BMI). Each factor adds points to the score, which ranges from 0 to ≥3, allowing the patient to be classified as low (0), intermediate (1–2), or high (≥3) risk for VTE. High-risk patients may be considered for thromboprophylaxis, according to clinical guidelines, always with individualized assessment. This score is recommended by ASCO for oncological practice [15]. However, it presents flaws that we believe can be remedied with the evaluation of cellular and molecular components. In this context, circulating tumor cells (CTCs) and circulating tumor microemboli (CTMs) emerge as elements of great interest [16].

CTCs are cells that detach from the primary tumor or metastatic sites and enter the bloodstream, with the ability to initiate new metastases in distant organs. Their presence in peripheral blood has been associated with a worse prognosis in various types of cancer [17]. More recently, increasing evidence suggests that CTCs are not only markers of tumor dissemination but also can play an active role in promoting thrombosis. It is believed that these cells can interact with platelets, leukocytes, and the coagulation system, contributing to thrombus formation. CTMs, which are aggregates of CTCs, may have an even greater thrombogenic potential due to their size and the concentration of procoagulant factors [18].

Given the clinical relevance of VTE in oncology and the growing understanding of the role of CTCs and CTMs, this review aimed to provide a comprehensive overview of the current literature on the relationship between thromboembolic events, CTCs, and CTMs. The pathophysiological mechanisms involved, the main molecular and cellular markers identified, the findings of relevant clinical studies, and the implications for clinical practice were addressed. The critical analysis of the available evidence aimed to highlight the prognostic importance and potential clinical applications of evaluating CTCs and CTMs in the context of cancer-associated VTE, as well as to point out directions for future research in this promising field.

## Pathophysiology of thromboembolic events in cancer

The pathophysiology of thromboembolic events in oncological patients represents a complex and multifactorial process, involving interactions between tumor cells, the hemostatic system, and the host’s inflammatory response [19]. Understanding these mechanisms is fundamental for the development of effective prevention and treatment strategies, as well as for the identification of biomarkers with prognostic and predictive value.

The classic Virchow’s triad, which includes venous stasis, endothelial injury, and hypercoagulability, remains a valid conceptual model for understanding the pathogenesis of VTE in the context of cancer [20]. However, oncological patients present peculiarities that amplify each of these components. Venous stasis can be exacerbated by direct tumor compression of vascular structures, prolonged immobilization due to advanced disease or treatment effects, and by hospitalization itself. Endothelial injury can result from direct tumor invasion, effects of chemotherapy and radiotherapy, or procedures such as central venous catheterization. However, it is in the hypercoagulability component that the most specific and complex mechanisms of the interaction between cancer and thrombosis reside [21].

Neoplastic cells can activate the coagulation system through multiple mechanisms. One of the most studied is the increased expression of tissue factor (TF), a transmembrane glycoprotein that initiates the extrinsic pathway of coagulation [22,23]. TF is constitutively expressed in many types of tumor cells, and its expression correlates with tumor aggressiveness and metastatic potential. When exposed to blood, TF forms a complex with activated factor VII (FVIIa), triggering an activation cascade that culminates in thrombin generation and fibrin formation. In addition to cell surface expression, tumor cells can release TF-containing microparticles, which circulate in the blood and contribute to the systemic hypercoagulable state [24–28].

Besides TF, tumor cells can secrete other procoagulant substances, such as cancer procoagulant (CP), a cysteine protease that directly activates factor X, independently of factor VII. Studies have shown elevated levels of CP in patients with various types of cancer, correlating with more advanced stages of the disease. Neoplastic cells can also interfere with the fibrinolytic system through the production of plasminogen activator inhibitors (PAI-1 and PAI-2), resulting in an imbalance that favors clot formation and persistence [29].

Inflammation plays a crucial role at the interface between cancer and thrombosis. Tumor cells secrete pro-inflammatory cytokines, such as interleukin-1β (IL-1β), tumor necrosis factor-α (TNF-α), and interleukin-6 (IL-6), which induce the expression of adhesion molecules on the vascular endothelium, promote platelet activation, and increase the hepatic synthesis of coagulation factors. This chronic inflammatory state significantly contributes to the pro-thrombotic phenotype observed in cancer patients [30–34].

In the context of CTCs and CTMs, increasing evidence suggests that these entities are not only markers of tumor dissemination but also active participants in the pathogenesis of VTE. CTCs express high levels of TF and other procoagulant proteins, which can directly initiate coagulation in the bloodstream. Furthermore, CTCs can interact with platelets, forming aggregates that facilitate both the survival of tumor cells in circulation and the formation of thrombi. This CTC–platelet interaction is mediated by various adhesion molecules, including P-selectin, integrins, and platelet surface glycoproteins [35–37].

CTMs, which consist of aggregates of CTCs, have an even greater thrombogenic potential due to the concentration of procoagulant factors and the larger contact surface with blood elements. Recent studies have shown that the presence of CTMs correlates with a higher risk of thromboembolic events in some types of cancer, although this association is not universal for all neoplasms [38]. The heterogeneity of the results may reflect methodological differences in CTM detection, biological variations among different cancer types, or the influence of uncontrolled confounding factors in the studies.

**Figure 1:**
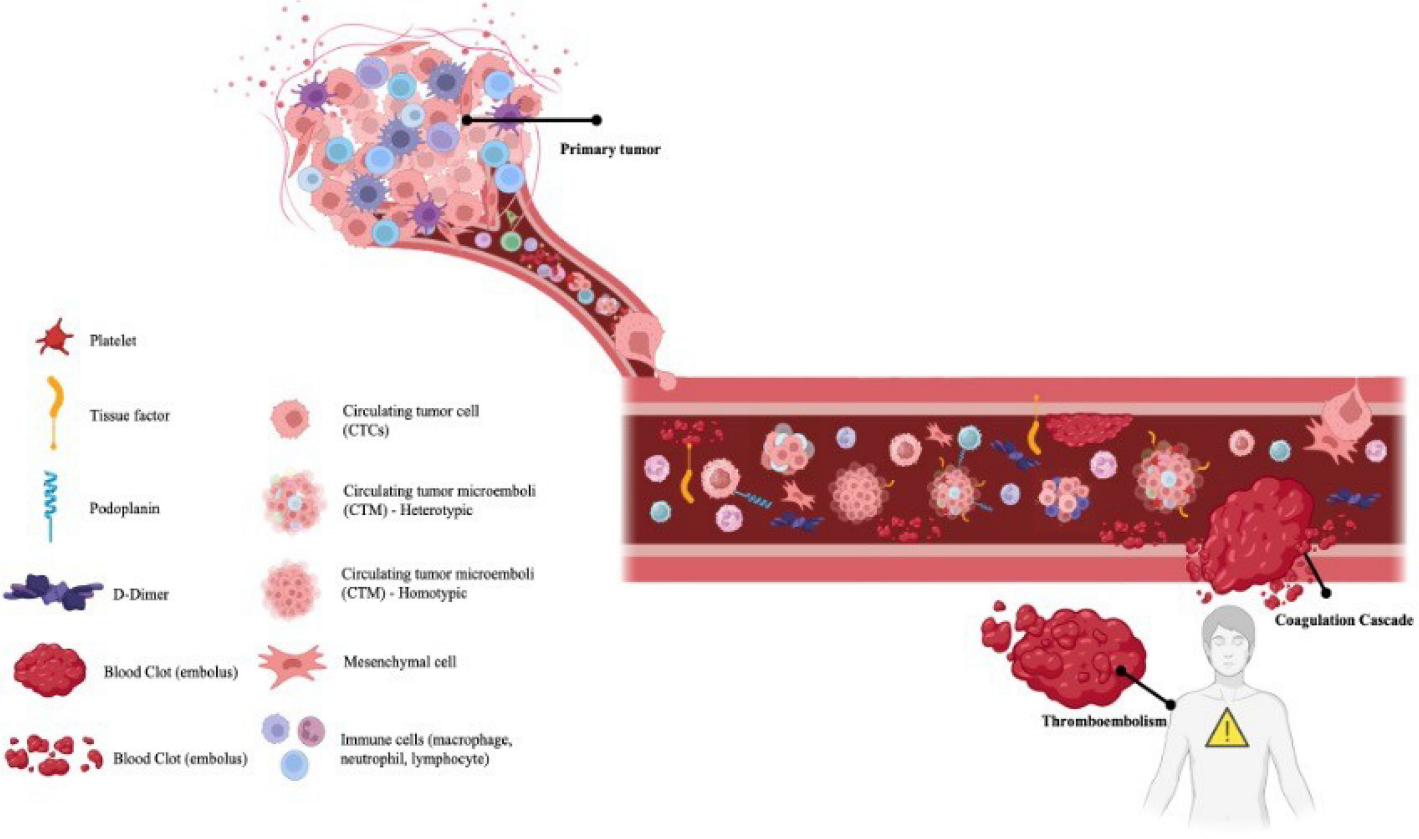
Schematic representation of tumor progression and associated thromboembolic events. Tumor cells detach from the primary tumor and enter the bloodstream (circulating tumor cells; CTCs), where they can form circulating tumor microemboli (CTMs), which can be homotypic (composed only of tumor cells) or heterotypic (associated with other cells, such as platelets and leukocytes). These structures interact with blood components such as platelets, tissue factor, podoplanin, and immune cells, creating an environment conducive to activation of the coagulation cascade. This process promotes thrombus formation and the development of thromboembolic events, often accompanied by elevated D-dimer levels. Furthermore, these interactions help to protect CTCs, facilitating immune evasion and potentially enabling metastasis to distant sites.

The platelet-to-lymphocyte ratio (PLR) has emerged as a promising biomarker at the interface between inflammation, cancer, and thrombosis. High PLR values reflect both a pro-inflammatory state (increased platelets) and a suppression of antitumor immunity (reduced lymphocytes). Clinical studies, such as that by Pignataro et al., demonstrated that a PLR >288 is significantly associated with a higher risk of VTE (*P*=0.005) and worse recurrence-free survival (*P*<0.0001) in patients with gastric cancer. This marker has the advantage of being easily obtained from routine blood counts, making it accessible in clinical practice [18] (Figure 1).

## Circulating tumor cells

CTCs are defined as neoplastic cells that detach from the primary tumor or metastatic sites and gain access to the bloodstream, constituting a crucial step in the process of metastatic dissemination. Their identification and characterization have revolutionized both the understanding of tumor progression and the development of prognostic biomarkers in oncology [39].

From a biological point of view, CTCs are not simply passive fragments of the original tumor, but cellular entities with specific characteristics that allow them to survive in the hostile environment of the bloodstream. To this end, these cells often undergo phenotypic changes, such as epithelial-mesenchymal transition (EMT), which confers greater plasticity, resistance to apoptosis, and invasive capacity. During this process, CTCs can acquire tumor stem cell properties, increasing their potential for tumor initiation and resistance to conventional therapies [40–42].

The detection and quantification of CTCs represent a considerable technical challenge, given their rarity in circulation and phenotypic heterogeneity. Several methods have been developed to isolate and characterize these cells, each with specific advantages and limitations. Approaches based on physical characteristics, such as size and deformability, include size-based filtration (such as the ISET TM - Isolation by SizE of Tumor cells, Rarecells, France) and density gradient centrifugation. Methods based on biological properties use cell surface markers, such as EpCAM (Epithelial Cell Adhesion Molecule) for positive capture (CellSearch® system, Menarini Biosystems) and CD45 for negative leukocyte depletion [43–50].

Recent advances in detection technologies include microfluidic systems (‘CTC chips’), which combine physical and biological principles to increase capture efficiency, and single-cell sequencing techniques, which allow detailed genomic and transcriptomic analyses of isolated CTCs. The molecular characterization of CTCs has revealed significant heterogeneity, even within the same patient, reflecting the clonal evolution of the tumor and potentially contributing to different biological behaviors, including thrombogenic potential [51–53].

The prognostic value of CTCs has been extensively documented in various types of cancer. In the multicenter study conducted by Iinuma et al., the detection of CTCs expressing specific markers (CEA, CK19, CK20, and/or CD133) in the peripheral blood of colorectal cancer patients with Dukes' B and C stages was associated with significantly worse overall and disease-free survival. Multivariate analysis confirmed that the presence of these CTCs constituted an independent prognostic factor, with a hazard ratio of 3.84 for overall survival (95% CI: 2.41–6.22; *P*<0.001) and 3.02 for disease-free survival (95% CI: 1.83–5.00; *P*<0.001). Similar results have been reported in breast cancer, prostate cancer, lung cancer, and other tumor types, consolidating CTCs as a robust prognostic biomarker [17].

A particularly interesting aspect is the possible relationship between specific CTC subpopulations and thrombotic risk. CTCs with a tumor stem cell phenotype, identified by the expression of markers such as CD133, may present a greater thrombogenic potential due to the increased expression of procoagulant factors. Similarly, CTCs that have undergone EMT, characterized by the loss of epithelial markers and gain of mesenchymal markers, can interact more efficiently with platelets and other blood elements, increasing the risk of thrombus formation [54–62].

The understanding of the relationship between CTCs and VTE has significant clinical implications. The detection of CTCs, especially those with phenotypes associated with greater thrombogenic potential, could potentially be incorporated into risk stratification models for VTE in cancer patients, complementing established scores such as the Khorana score. Furthermore, interventions targeting CTCs or their interactions with the hemostatic system could represent innovative strategies for VTE prevention in this high-risk population.

In conclusion, CTCs represent not only a prognostic biomarker in oncology but also a potentially important element in the pathogenesis of cancer-associated VTE.

## Circulating tumor microemboli

CTMs, also referred to as CTC clusters, are defined as aggregates or clusters containing three or more CTCs with distinct nuclei. CTMs have garnered increasing scientific interest due to their role in metastatic progression and possible contribution to thromboembolic events in cancer patients [63].

The formation of CTMs can occur through different mechanisms. The most common involves the collective detachment of tumor cells from the primary tumor or metastatic sites, preserving the original intercellular junctions. Alternatively, isolated CTCs can aggregate in the bloodstream, often with the participation of platelets, leukocytes, and extracellular matrix components. This heterogeneity in the composition of CTMs contributes to their biological complexity and potential clinical impact [64–66].

From a biological perspective, CTMs exhibit characteristics that distinguish them from isolated CTCs. Experimental studies have shown that CTMs have greater metastatic efficiency, with up to 50 times higher capacity to form colonies in animal models. This advantage can be explained by several factors: protection against anchorage-induced apoptosis (anoikis), resistance to hemodynamic stress in circulation, evasion of immunological surveillance, and greater efficiency in extravasation and colonization of distant organs. Furthermore, cellular heterogeneity within CTMs can promote co-operation between different subpopulations of tumor cells, enhancing their survival and proliferation capacity [67]. See details in Figure 1.

The detection and characterization of CTMs present even greater technical challenges than those faced for isolated CTCs. Filtration-based methods, such as the ISET system, have proven particularly suitable for the identification of CTMs, as they preserve the integrity of cellular aggregates [68]. In contrast, technologies based on final reading in a flow cytometry system, such as the CellSearch® system, may be less efficient in detecting CTMs, as they lyse the aggregates for passage through the tubes [43]. Recent advances include microfluidic platforms specifically designed to capture and preserve CTMs, allowing for more detailed molecular and functional analyses [69].

The prevalence of CTMs varies significantly among different cancer types and disease stages. In the study by Pignataro et al. with gastric cancer patients, CTMs were detected in approximately 39.8% of cases [38]. Other studies reported prevalences of CTMs, along with CTCs, that vary significantly depending on the type of cancer, disease stage, and detection methodology. In general, the presence of CTMs is more commonly associated with advanced stages of disease and poorer prognosis, although this correlation is not universally observed across all tumor types [70–74].

Clinical studies specifically investigating the association between CTMs and VTE in cancer patients are still limited and show heterogeneous results. Vona et al. [75] evaluated peripheral blood (6 ml) of 44 patients with primary liver cancer without metastases and observed that the presence of CTCs/CTM was more frequent in patients with diffuse tumors (*P*=0 .0001). CTCs/CTMs were also associated with portal tumor thrombosis (*P*=0.006). The authors analyzed the relationship between CTCs/CTM in blood and the histological evidence of microinvasion of cancer cells in vessels in 18/22 patients that were submitted to liver tumor resection. Tumors spread into the venous vessels around the tumor tissue in 6/18 patients. Curiously, among those six, five had CTCs/CTM in the peripheral blood. This result points to the association between portal tumor thrombosis and presence of CTCs/CTM [75]. In the study by Pignataro et al., VTE developed in 7/37 (18.9%) CTM-positive patients and in 11/50 (22%) CTM-negative patients (*P*=0.93), showing no statistically significant association.

This heterogeneity of results can be explained by several factors. First, methodological differences in the detection and characterization of CTMs can significantly influence the findings [76]. Furthermore, the composition and biological properties of CTMs can vary among different cancer types, resulting in distinct thrombogenic potentials. Finally, the interaction between CTMs and the hemostatic system is likely modulated by patient-specific factors, such as comorbidities, concomitant treatments, and genetic polymorphisms related to coagulation (Table 1).

**Table 1:** Methods for CTCs/CTM evaluation and correlation of CTCs/CTM with VTE in oncological patients

Detection method	Technique principle	Markers used	Cancer type	VTE findings	References
Immunomagnetic capture + RT-PCR	Combination of techniques	Multiple (EpCAM, MUC1, HER2)	Breast	Detectable CTCs associated with higher VTE risk (OR 5.29, 95% CI 1.58–17.7, *P*=0.007).	[77]
CellSearch® (Menarini Biosystems)	Flow cytometry	EpCAM, cytokeratins (CK8, CK18, CK19), CD45 (negative)	Prostate	In 28 patients with refractory prostate cancer, PSA changes correlated with CTC (*r* = 0.67) and D-dimer (*r* = 0.58), and CTC with D-dimer (*r* = 0.62; all *P*<.001). Increases in PSA and D-dimer, but not CTCs, were associated with progressive disease (*P*<.024).	[78]
Real-time RT-PCR	Detection of mRNA of specific markers	CEA, CK19, CK20, CD133	Colorectal (Dukes' B and C)	VTE was not directly evaluated, but CTC + patients showed worse overall and disease-free survival.	[17]
CellSearch® (Menarini Biosystems)	EpCAM-based immunomagnetic capture followed by flow cytometry characterization	EpCAM, cytokeratins (CK8, CK18, CK19), CD45 (negative)	Metastatic breast	In multivariate analysis, CTCs + and number of metastases were positively associated with plasma D-dimer level. At median follow-up of 13.5 months, 3/33 patients (9 %) with ≥ 1 CTC had VTE, vs. no patients with undetectable CTCs.	[79]
OncoBean Chip	Microfluidic device for CTC capture	EpCAM, EGFR, CD133	Lung	VTE was not assessed; CTC clusters correlated with stage and trended toward shorter PFS, suggesting clinical relevance.	[80]
CellSearch® (Menarini Biosystems)	Flow cytometry	EpCAM, citoqueratins (CK8, CK18, CK19), CD45 (negative)	Metastatic breast	Multivariate analysis including age, Khorana score, baseline lactate dehydrogenase, and CTC levels showed that detectable CTC was the only factor significantly associated with an increased risk of TE (SHR* for patients with [1–4] CTC = 3.1, 95% CI [1.1; 8.6], SHR for patients with ≥ 5 CTC = 1.4, 95% CI [0.5; 4.6]). *SHR = Subdistribution hazard ratio	[81]
Negative enrichment techniques	Depletion of CD45+cells followed by cellular characterization	CD45 (negative), EpCAM, cytokeratins positive	Various	Review: Greater ability to detect CTCs with EMT phenotype, potentially more thrombogenic	[82]
Negative enrichment	Negative separation (immunomagnetic beads) of CD45+cells	CD45 negative; Pan-CK	Lung	The incidence of D-Dimer, platelets, and distant metastasis was significantly higher in the CTC + patient group compared with the CTC- group (*P*<0.05).	[83]
CellSearch® (Menarini Biosystems)	Flow cytometry	EpCAM, citoqueratins (CK8, CK18, CK19), CD45 (negative)	Bladder	CTCs were detected in 22.8% of patients (43 out of 189). VTE was experienced in 6 patients (3.2%) and 8 patients (4.2%) had cardiovascular events (CVE) in the postoperative period.	[84]
ISET (Isolation by SizE, Rarecells)	Size-based filtration (8 μm pores)	Cell morphology	Gastric	VTE in 18.9% of CTM + patients vs. 22% of CTM- (*P*=0.93). No significant correlation. When the platelet-to-lymphocyte ratio (PLR) > 288, VTE was present in 7/14 patients (*P*=0.005). PLR is also associated with poor recurrence-free survival, as CTCs at the follow-up (*P*<0.0001).	[38]

A particularly interesting aspect is the possible interaction between CTMs and other risk factors for VTE in cancer patients. For example, the combination of CTMs with elevated inflammatory markers or an increased PLR could potentially identify a subgroup of patients with a particularly high risk of thromboembolic events. This integrated approach, considering multiple biomarkers, could improve risk stratification and guide therapeutic decisions related to thromboprophylaxis [85].

The clinical implications of CTM detection in the context of cancer-associated VTE are still being elucidated. If confirmed by robust prospective studies, the association between CTMs and an increased risk of VTE could justify the incorporation of this biomarker into risk stratification models. Furthermore, interventions targeting CTMs or their mechanisms of interaction with the hemostatic system could represent innovative strategies for VTE prevention in high-risk cancer patients.

## Molecular and cellular markers

The identification and characterization of molecular and cellular markers related to thromboembolic events, CTCs, and CTMs not only contribute to the understanding of the underlying pathophysiological mechanisms but also offer potential for clinical application in risk stratification, disease monitoring, and the development of targeted therapies.

Among the most studied molecular markers in this context, tissue factor (TF) holds a prominent position. This transmembrane glycoprotein, the main initiator of the extrinsic coagulation pathway, is constitutively expressed in many types of tumor cells, and its expression often correlates with tumor aggressiveness and metastatic potential. Studies have shown that TF-positive CTCs can promote thrombin generation and fibrin formation directly in the bloodstream, establishing a mechanistic link between these cells and thromboembolic events. In addition to cell surface expression, TF can also be released in tumor-derived microparticles, which circulate in the blood and contribute to the systemic hypercoagulable state observed in cancer patients [86–88]

P-selectin, an adhesion molecule expressed by activated platelets and endothelial cells, represents another relevant marker at the interface between cancer and thrombosis. This glycoprotein mediates interactions between tumor cells, platelets, and leukocytes, facilitating the formation of aggregates that can contribute to both metastatic dissemination and thromboembolic events. Elevated levels of soluble P-selectin have been associated with a higher risk of VTE in cancer patients, suggesting its potential as a predictive biomarker [89–91].

D-dimer, a fibrin degradation product, is widely used as a marker of coagulation and fibrinolysis activation. In cancer patients, elevated D-dimer levels have been consistently associated with a higher risk of VTE and worse prognosis. However, its specificity is limited, as it can be elevated in various conditions, including inflammation, infection, and postoperative states. Despite this limitation, D-dimer remains an important component in risk stratification models for VTE in cancer patients [92,93].

Podoplanin (PDPN) is a transmembrane glycoprotein involved in physiological processes such as the development of the lymphatic system, platelet production in the bone marrow, and immune response. Its main interaction occurs with the CLEC-2 receptor, present on platelets, activating them and promoting platelet aggregation. This mechanism is physiologically essential, but, in the tumor context, it favors the formation of microthrombi and contributes to metastatic dissemination. Its expression promotes thrombosis by activating platelets and favoring the formation of metastatic niches protected by platelet aggregates, in addition to increasing the risk of VTE in cancer patients [94–97].

Thus, the podoplanin-CLEC-2 pathway represents not only a prognostic biomarker but also an emerging therapeutic target, with the potential to prevent thrombotic and metastatic events in cancer [98]. In cancer, podoplanin is frequently overexpressed in tumor cells of glioblastomas, head and neck carcinomas, and mesotheliomas, being associated with poor prognosis [99]. It is known that CTCs are capable of activating platelets; thus, although little explored, the analysis of podoplanin in CTCs can be an important tool for monitoring thromboembolic events in cancer patients.

In this context, the evaluation of CTCs and CTMs gains relevance as potential biomarkers of thrombotic and metastatic risk. A recent study assessed the clinical implications of CTCs and CTMs in patients with gastric adenocarcinoma, with a particular focus on plakoglobin expression. Among 55 patients, plakoglobin expression was analyzed in 47 and detected in 59.6% of baseline CTCs; however, no significant association was found with progression-free survival (PFS). Notably, plakoglobin expression in CTMs was clinically relevant. Its presence in CTMs, but not in isolated CTCs, was associated with poorer prognosis and a trend toward reduced PFS. Moreover, the combined expression of plakoglobin in CTMs or HER2 in CTCs was identified as a strong predictor of poor progression-free survival (*P*=0.027), particularly in patients with the diffuse histological subtype of gastric cancer [100] These findings reinforce the importance of characterizing both individual and clustered CTCs, as well as their molecular markers, such as podoplanin and plakoglobin, to better stratify thrombotic and metastatic risks in oncology.

CD133 deserves special attention, as it is a marker of circulating tumor stem cells, a subpopulation of CTCs with a greater capacity for self-renewal, differentiation, and tumor initiation. Studies suggest that CD133-positive CTCs may present a greater thrombogenic potential due to the increased expression of procoagulant factors, establishing a possible link between circulating tumor stem cells and the risk of VTE [101,102]

A small noncoding RNA molecule, such as MIR-203A-3P, and matrix metalloproteinase-2 (MMP-2) have been identified as highly expressed in CTCs from pancreatic carcinoma patients. MMP-2 plays a crucial role in extracellular matrix degradation, facilitating tumor invasion and metastasis. Furthermore, studies suggest that MMPs can interact with the hemostatic system, potentially contributing to the hypercoagulable state in cancer patients [103]

Markers related to platelet activation have also been investigated in the context of CTCs, CTMs, and thromboembolic events. Platelets play a crucial role in CTC biology by forming protective ‘coatings’ that shield them from immune system clearance and facilitate their adhesion to the vascular endothelium. Platelet activation markers such as CD62P (P-selectin), CD63, and platelet factor 4 (PF4) may potentially reflect these interactions and correlate with the risk of VTE in patients with detectable CTCs [104–106].

**Figure 2:**
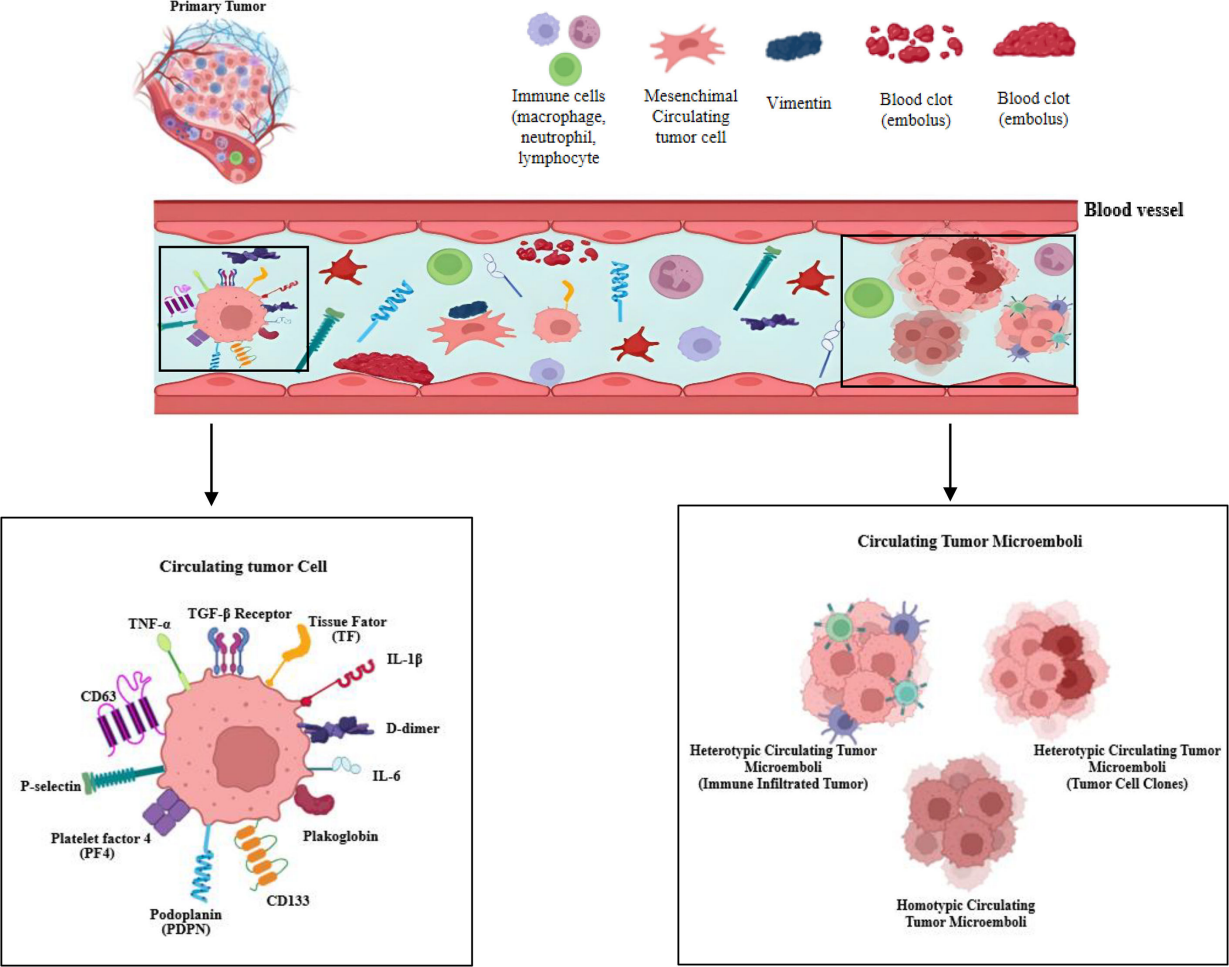
Schematic representation of the release of circulating tumor cells (CTCs) and circulating tumor microemboli (CTMs) from the primary tumor, along with their key cellular and molecular components. The upper panel illustrates the primary tumor and the entry of various cellular and extracellular elements into the blood vessel, including immune cells (macrophages, neutrophils, lymphocytes), platelets, mesenchymal circulating cells, structural proteins such as vimentin, and blood clots (emboli). Within the vascular compartment, CTCs and CTMs interact with immune cells, platelets, and additional components of the circulating microenvironment. The lower left panel highlights the molecular composition of a circulating tumor cell (CTC), demonstrating markers associated with inflammation, endothelial activation, and thrombogenicity, including TNF-α, TGF-β receptor, tissue factor (TF), IL-1β, IL-6, D-dimer, CD63, P-selectin, platelet factor 4 (PF4), podoplanin (PDPN), plakoglobin, and CD133

Inflammatory cytokines such as interleukin-6 (IL-6), tumor necrosis factor-alpha (TNF-α), and interleukin-1 beta (IL-1β) represent another relevant group of biomarkers. These molecules not only promote a prothrombotic state through the induction of coagulation factors and inhibition of fibrinolysis but may also influence CTC biology by enhancing their survival and metastatic potential. Elevated levels of these cytokines have been associated with both an increased risk of VTE and a poorer prognosis in various cancer types [107–109].(Figure 2)

The lower right panel presents the different forms of CTMs, including: heterotypic CTMs infiltrated by immune cells, heterotypic CTMs composed of distinct tumor cell clones, homotypic CTMs, consisting exclusively of tumor cells. These structures contribute critically to metastatic dissemination and to the establishment of a systemic prothrombotic state.

Tumor-derived microparticles constitute a heterogeneous group of extracellular vesicles released by tumor cells, capable of carrying a variety of bioactive molecules, including tissue factor (TF), microRNAs, and signaling proteins. These microparticles can directly activate the coagulation cascade and modulate the function of platelets, leukocytes, and endothelial cells, thereby contributing to a hypercoagulable state. The quantification and characterization of TF-positive microparticles have been proposed as potential biomarkers for VTE risk in cancer patients [110].

The integration of multiple markers into risk panels or scoring systems represents a promising strategy to improve the stratification of cancer patients regarding VTE risk. For example, combining traditional markers such as D-dimer and platelet count with more specific markers—such as tissue factor (TF)-positive CTCs and RPL expression, or the presence of CTMs—could potentially identify subgroups of patients at particularly high risk who may benefit from more intensive thromboprophylaxis. For more examples, see Table 2.

**Table 2:** Incidence and characteristics of VTE in different cancer types

Cancer type	VTE incidence	Specific risk factors	Prognostic impact	Associated biomarkers	References
Pancreatic	Up to 8.1% to 12% over 1–2 years	Pancreatic adenocarcinoma, surgery, chemotherapy, advanced stage	VTE associated with higher mortality and complications	D-dimer, P-selectin, tissue factor microparticles	[111,112]
Lung	~5.1% in hospitalized patients	Adenocarcinoma, chemotherapy, metastases	VTE worsens overall survival	D-dimer, tissue factor-positive microparticles	[113]
Gastric	4.9% to 15%	Advanced stage, adenocarcinoma	VTE correlates with worse prognosis	Inflammatory platelet relationships (e.g. platelet-to-lymphocyte ratio)	[38,112]
Ovarian	5.6% up to 14–27% within 1 year	Surgery, platinum-based chemotherapy, ascites	VTE may reduce survival, especially in advanced stages	Elevated platelet count, CA-125	[114–116]
Brain (glioblastoma)	~4.8% per year	Neurosurgery, immobility, high-grade tumor	VTE associated with reduced survival	P-selectin, D-dimer, GDF-15 (Growth Differentiation Factor-15)	[117,118]
Hematologic malignancies	3–5%	Chemotherapy, thalidomide/lenalidomide, leukocytosis	VTE delays treatment and worsens prognosis	Leukocytosis, P-selectin	[112,119]
Colorectal	~3%	Central venous catheter, surgery, chemotherapy, advanced stage	VTE associated with higher mortality	Elevated CEA, D-dimer	[112,113]
Breast	2–5% in chemotherapy/hormonal therapy	Chemotherapy, hormonal therapy (tamoxifen), metastases	VTE reduces survival by 2–3 times	CTCs, D-dimer	[112,120]
Prostate	< 2–3%	Androgen deprivation therapy, bone metastases	Variable impact	Elevated PSA, D-dimer	[112,113]

Advances in molecular analysis technologies, including next-generation sequencing, proteomics, and metabolomics, are enabling the discovery of novel biomarkers with potential clinical application in this field. Detailed molecular characterization of CTCs and CTMs, including gene expression profiles, epigenetic alterations, and proteomic signatures, may uncover new markers with greater specificity and predictive value for thromboembolic events in cancer patients.

## Clinical studies

The study by Pignataro et al. [18] investigated the relationship between the PLR, CTCs, and VTE in patients with gastric cancer. This observational study included 93 patients with localized and metastatic gastric cancer. CTCs were analyzed using the ISET platform at two time points: before neoadjuvant treatment (CTC1) and after surgery/before adjuvant therapy (CTC2) for patients with localized disease; or before first-line chemotherapy (CTC1) and after 6 months (CTC2) for patients with metastatic disease. The incidence of VTE was determined retrospectively. The results showed that 63 patients (67.7%) were at intermediate risk and 30 (32.3%) at high risk for VTE according to the Khorana score. The overall incidence of VTE was 20.4%, which is consistent with the literature for this cancer type. CTMs were found in 39.8% of patients, indicating a considerable prevalence. Surprisingly, VTE occurred at similar proportions among patients positive for CTMs (7/37, 18.9%) and negative for CTMs (11/50, 22%), with no statistically significant difference (*P*=0.93). This finding contrasts with the initial hypothesis that CTMs would be associated with an increased risk of VTE, suggesting that the relationship between these entities may be more complex than previously assumed [35]

On the other hand, the same study found a significant association between elevated PLR and VTE risk. When PLR was greater than 288, VTE occurred in 7 out of 14 patients (*P*=0.005), suggesting that this simple hematological parameter may serve as a more robust predictor of thromboembolic events than the presence of CTCs or CTMs in this specific population. Moreover, elevated PLR was also associated with worse recurrence-free survival (*P*<0.0001), reinforcing its overall prognostic value. A particularly noteworthy aspect of the study by Pignataro et al. was the multivariate analysis, which identified PLR, CTC2, and VTE as independent prognostic factors for recurrence-free survival. This finding suggests that although the presence of CTMs was not directly correlated with VTE risk, both CTCs and thromboembolic events represent important prognostic markers in patients with gastric cancer [76]

Other studies have investigated the relationship between specific markers expressed by CTCs and the risk of VTE. Tormoen et al. demonstrated that tissue factor (TF)-positive CTCs can promote coagulation in vitro and may potentially contribute to a hypercoagulable state *in vivo*. In a study involving patients with pancreatic cancer, elevated levels of TF expression in CTCs were correlated with an increased risk of thromboembolic events, suggesting that not only the presence of CTCs, but also their molecular profile, may influence thrombotic risk [121]

The study conducted by Bourcy et al. [122] demonstrated, through *in vitro* experiments, animal models, and analysis of patients with metastatic breast cancer, that CTCs undergoing epithelial–mesenchymal transition (EMT)—a process that grants epithelial CTCs enhanced migratory, invasive, and survival capabilities, thus promoting metastasis—are associated with coagulation and poorer survival. This study showed that EMT induces the expression of TF, a key activator of the coagulation cascade, as previously discussed. In vitro assays using EMT-positive breast cancer cell lines (MDA-MB-231 and Hs578T), EMT-negative lines (MCF7 and T47D), and cell lines capable of EMT induction (MDA-MB-468, A549, and PMC42-LA), analyzed by qRT-PCR and Western blotting, revealed high TF expression in EMT-positive lines (characterized by high vimentin and low E-cadherin expression). TF was overexpressed at both the mRNA and protein levels in all EMT-induced lines. Flow cytometry confirmed these findings, showing significantly higher TF expression in EMT-positive cells compared with other lines. Furthermore, visual clotting assays and thromboelastometry demonstrated that clot formation time was shorter in the presence of EMT + cells with high TF expression than with EMT- cells. In animal models, EMT + cells could initiate metastatic steps, and the involvement of TF in this process enhanced their resistance and metastatic potential. To establish a direct link between EMT, metastasis, and coagulation, mice injected with EMT + cells were pre-treated with anticoagulants, resulting in reduced lung colonization. Another important observation was that lung-colonizing cells were surrounded by microthrombi, platelets, and fibrin. To validate the experimental findings, CTCs were enriched from blood samples of patients with metastatic breast cancer (*n* = 22) using the ScreenCell filtration method, followed by triple fluorescent staining (pan-cytokeratins, TF, and vimentin). CTCs expressing cytokeratins, TF, and vimentin were identified in 19 of 22 patients (86.3%). CTCs positive for cytokeratins and TF only were observed in 81.8% of patients. In contrast, CTCs expressing cytokeratins and vimentin without TF were found in only 9 patients (40.9%).

Importantly, among patients with more than three CTCs co-expressing TF and vimentin, overall survival (OS) was significantly shorter (*P*=0.039). Although limited by sample size, this study supports the hypothesis that increased TF expression may promote local coagulation, leading to the entrapment of EMT + CTCs within platelet/fibrin microthrombi, thereby facilitating their survival and early dissemination to colonized organs [122].

The prognostic value of podoplanin (PDPN) expression in CTCs was investigated by Hsieh et al. [123]. This study included 53 patients with head and neck squamous cell carcinoma (HNSCC). CTCs were enriched through negative selection of leukocytes followed by immunofluorescent staining for EpCAM and PDPN. In this context, the ratio of PDPN^+^ to EpCAM^+^ CTCs (PDPN^+^/EpCAM^+^-CTCs) was shown to be a significant prognostic marker in patients with HNSCC. Receiver operating characteristic (ROC) analysis indicated that this ratio had a better predictive performance for 6 month mortality (AUC = 0.718; *P*=0.011) than either the absolute number of CTCs or PDPN^+^-CTCs alone. Univariate analysis showed that a PDPN^+^/EpCAM^+^ ratio greater than 20% was significantly associated with poorer progression-free survival (HR = 4.666; *P*=0.016) and overall survival (HR = 12.400; *P*=0.015). These findings were confirmed in multivariate analysis, where the ratio remained an independent prognostic factor for overall survival (HR = 10.969; *P*=0.018). Kaplan–Meier curves further demonstrated that only the PDPN^+^/EpCAM^+^-CTC ratio—not the absolute count of total CTCs or PDPN^+^-CTCs—was able to significantly discriminate between patients with favorable prognosis ( ≤ 20%) and those with worse clinical outcomes (>20%) [123].

## Therapeutic implications

Risk stratification for VTE in cancer patients is one of the most immediate clinical applications of the findings discussed in this review. Predictive models such as the Khorana score have been widely used to identify patients at high risk of VTE, thereby guiding decisions regarding thromboprophylaxis. Two modified versions of the Khorana score have been proposed. Besides the five predictive variables of the original score, the Vienna Cancer and Thrombosis Study (CATS) score included elevated levels of soluble P-selectin (1 point if ≥ 53.1 ng/ml) and elevated D-dimer levels (1 point if ≥ 1.44 μg/ml) at baseline ([117]). The Protecht score added: antineoplastic treatment (1 point if the regimen included cisplatin/carboplatin or gemcitabine-based chemotherapy; 2 points if both) [124]). Another score has been proposed to predict VTE after the start of antineoplastic treatment: the COMPASS-CAT, which includes cancer-related risk factors (specific type of treatment, time since diagnosis, presence of central venous catheter, cancer stage), predisposing factors (cardiovascular risk factors, recent hospitalization, personal history of VTE) and biomarkers (e.g.:platelet count) ([125]). The performance of these predictive scores has recently been questioned. A meta-analysis reported that the incidence of CAT, according to the Khorana score, was 5.0% in the low-risk group, 6.6% in the intermediate-risk group, and 11.0% in the high-risk group, which suggests that a high number of VTE events still occur in the low- and intermediate-risk groups [126]. So, the incorporation of novel biomarkers—such as the PLR, CTCs, and specific molecular markers—could potentially improve the accuracy of these predictive tools [127].

The implementation of biomarker-driven thromboprophylaxis strategies represents another relevant clinical implication. Current guidelines recommend pharmacological thromboprophylaxis for hospitalized patients with active cancer and for selected high-risk outpatients, typically defined according to the Khorana score. The identification of patients with elevated PLR, TF-positive CTCs, or other high-risk markers could support a more aggressive prophylactic approach, potentially including anticoagulation in ambulatory patients who would not ordinarily be considered candidates under existing criteria [128].

The optimal duration of anticoagulation in cancer patients with VTE remains a matter of debate. Current guidelines generally recommend extended anticoagulation (at least six months) for patients with active cancer and confirmed VTE [15]. However, due to the heterogeneity of this population, determining the ideal duration for each individual remains challenging.

The choice of anticoagulant agents may also be influenced by specific biomarkers. Recent studies have demonstrated the efficacy and safety of direct oral anticoagulants (DOACs) in cancer patients with VTE, offering an alternative to low-molecular-weight heparins (LMWHs) [129]. DOACs present some advantages in comparison with LMWHs: allow simple dosing schedules, have minimal known drug interactions, lack of necessity for regular monitoring, and allow dosage adjustment [130]. However, the heterogeneity of the oncologic population suggests that not all patients derive equal benefit from all agents.

Factor XI (FXI) inhibitors have emerged as potential safer alternatives to DOACs for the management of CAT ([130]). Pathological thrombosis can be triggered by two main pathways: the tissue factor pathway and the contact pathway. In both, thrombin is generated during the initial phase and subsequently activates Factor XI, which amplifies thrombin generation, promoting thrombus expansion and stabilization. FXI is considered essential for thrombosis, but not essential for hemostasis. Therefore, FXI inhibitors have the potential to reduce thrombosis with minimal or no disruption of hemostasis ([131]).

Abelacimab is a monoclonal antibody that binds FXI. If established, it could improve the main unmet needs in CAT: the parenteral administration could avoid the potential GI bleedings, its long half-life could allow the monthly dosage, its metabolism could favor cancer patients with severe kidney or liver failure [132]

The optimal duration of anticoagulant therapy for the secondary prevention of recurrent CAT remains uncertain, with limited evidence available. Current guidelines suggest indefinite anticoagulation while patients have persistent underlying risk factors (active or metastatic cancer, ongoing anticancer treatments) [129].

The decision to extend anticoagulation therapy (i.e., beyond the initial six months) for secondary prevention should be made on a case-by-case basis after assessing the risks and benefits of anticoagulant therapy. For patients requiring long-term anticoagulant therapy, the guidelines recommend the use of low-molecular-weight heparin (LMWH) or direct oral anticoagulants (DOACs) ([129]).

Persistent detection of CTCs or elevated PLR after the initial treatment period could potentially identify patients who would benefit from prolonged anticoagulation, while those lacking these risk markers might be candidates for earlier discontinuation, thereby reducing the risk of bleeding complications. Serial detection of CTCs may not only provide insights into disease progression and treatment response, but also serve as an early warning for increased risk of thromboembolic complications. This approach would allow for dynamic adjustments in thromboprophylaxis strategies throughout the course of the disease, adapting to changes in the patient’s individual risk profile. In addition, molecular characterization of CTCs and evaluation of specific biomarkers could potentially guide the personalized selection of anticoagulant therapy, optimizing the balance between efficacy and safety [133].

 The clinical implementation of these approaches faces significant challenges. The detection and characterization of CTCs require specialized technologies that are not yet widely available in routine clinical practice. Furthermore, methodological standardization and validation in prospective multicenter studies are necessary before these biomarkers can be incorporated into clinical guidelines. Cost is also a major barrier, although technological advances and economies of scale may eventually make these analyses more accessible.

Educating healthcare professionals about the importance of VTE in cancer patients and the potential value of novel biomarkers is another crucial aspect of clinical implementation. Studies have shown that VTE in cancer patients is often underdiagnosed and undertreated, highlighting the need for greater awareness of this potentially fatal complication. Disseminating knowledge about the relationship between CTCs, CTMs, and thromboembolic events could support a more proactive approach to the prevention and management of VTE in this population.

Understanding the relationship between thromboembolic events, CTCs, and CTMs offers multiple opportunities to improve the clinical management of cancer patients. The incorporation of biomarkers such as the PLR and CTCs into risk stratification models, the implementation of personalized thromboprophylaxis strategies, dynamic monitoring of thrombotic risk, and individualized selection of anticoagulant agents represent promising clinical applications of this knowledge. Despite implementation challenges, these approaches have the potential to significantly reduce the morbidity and mortality associated with VTE in cancer patients, ultimately contributing to improved overall outcomes in this population.

## Limitations of this review

**Figure 3:**
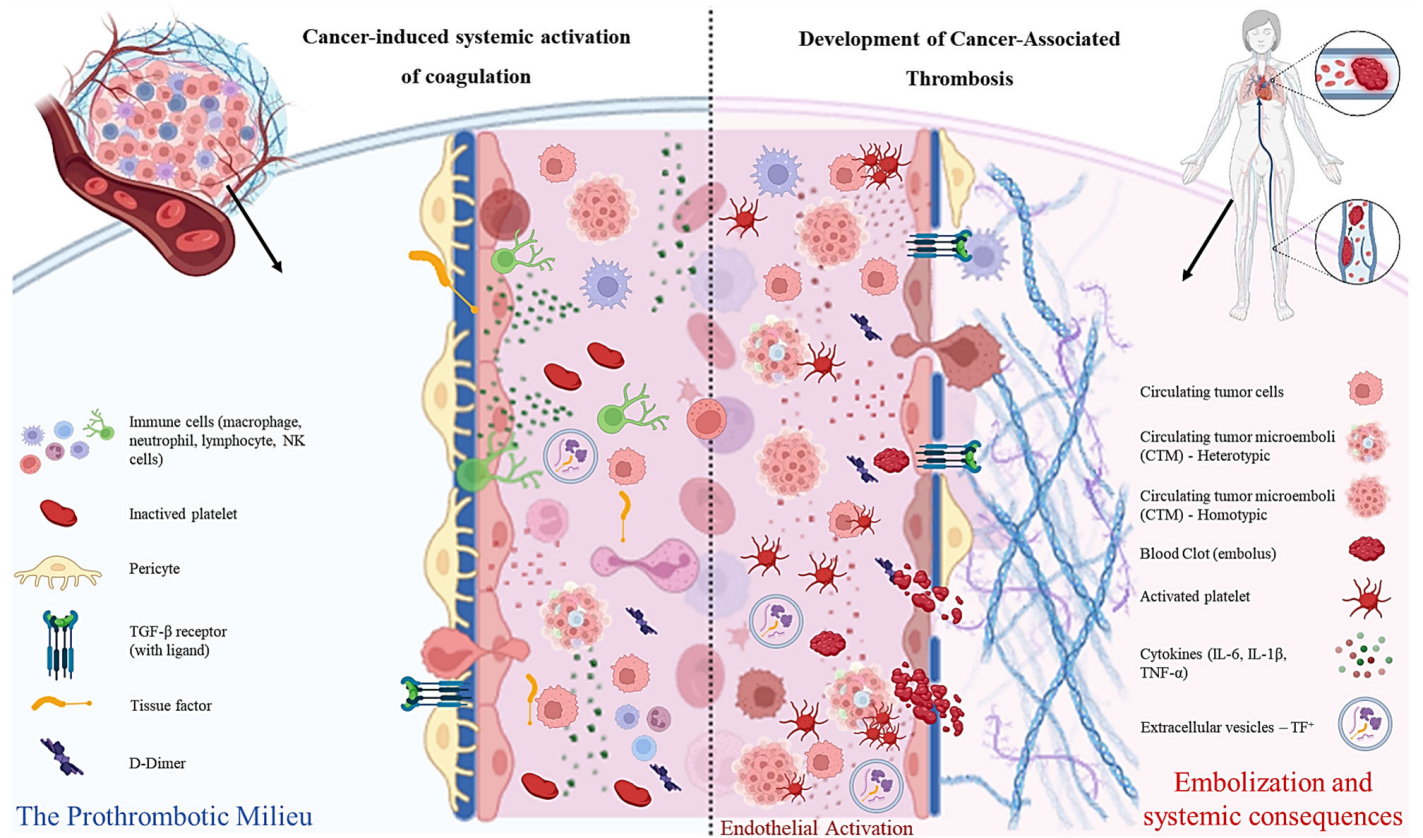
Cellular and molecular mechanisms underlying cancer-induced systemic activation of coagulation and the pathogenesis of cancer-associated thrombosis. The left panel depicts the prothrombotic milieu generated by the tumor, characterized by the expression and release of tissue factor (TF) by tumor cells and TF-bearing extracellular vesicles, thereby initiating the extrinsic coagulation pathway. Immune components of the tumor microenvironment—including macrophages, neutrophils, lymphocytes and NK cells—produce proinflammatory cytokines such as IL-6, IL-1β and TNF-α, which promote endothelial activation and up-regulation of procoagulant mediators. Pericytes, Circulating tumor cells (CTCs) and endothelial cells express TGF-β receptors, whose activation contributes to endothelial dysfunction and vascular remodeling. Circulating inactivated platelets are present within this environment and can be rapidly recruited in response to inflammatory stimuli. Elevated D-dimer levels reflect active fibrin formation and degradation. The right panel illustrates the progression to cancer-associated thrombosis. Endothelial activation enhances vascular permeability and the expression of adhesion molecules, facilitating interactions between tumor cells and the vascular wall. CTCs and circulating tumor microemboli (CTMs)—both heterotypic (containing immune cells and/or platelets) and homotypic (composed exclusively of tumor cells)—serve as niduses for thrombus initiation and amplification. Persistent release of cytokines and TF-positive extracellular vesicles sustains the hypercoagulable state. Activated platelets aggregate around CTCs and CTMs, fostering immune evasion and the development of fibrin-rich thrombi. Thrombus maturation ultimately results in embolization, leading to systemic clinical consequences, including venous thromboembolism and distal organ involvement.

The focus of our review was on CTCs and CTMs; therefore, we did not address a very important blood component involved in VTE formation: extracellular vesicles (EVs). EVs are composed of a lipid bilayer that can originate from the plasma membrane, forming large EVs, or be released from internal multivesicular bodies, forming small EVs. Hypercoagulability can be induced by EVs released by platelets, endothelial cells, and tumor cells through the expression of platelet receptors and the release of soluble agonists ([134]). CTCs can activate platelets and release EVs that, in turn, promote thrombosis, contributing to cancer progression [135]. As described in this review, CTCs also express platelet-associated receptors, including αIIbβ3, αVβ3, and GP-Ibα, promoting thrombus formation [136]. EVs derived from CTCs and endothelial cells initiate the extrinsic pathway of the coagulation cascade, generating thrombin along with podoplanin, which binds to CLEC-2 on platelets, triggering an activation response [137]. EVs derived from platelets can promote thrombosis through positive feedback loops of platelet activation, binding and activating neutrophils leading to the release of neutrophil extracellular traps (NETs), and intercellular communication with immune cells [138]. (Figure 3)

EVs of platelet, endothelial, and tumor origin are highly procoagulant, expressing tissue factor, phospholipids, platelet receptors, and internalized soluble mediators, creating a hypercoagulable environment in cancer patients [134].

Platelet activation, extracellular vesicle (EV) signaling, inflammation, and cancer progression are deeply interconnected; therefore, it is essential to investigate whether anticoagulants, particularly DOACs, influence EVs, CTCs, and CTM- and inflammation-mediated processes in unexpected ways, in order to provide clinicians with safety assurance beyond the already known advantages regarding VTE and bleeding rates.

## Open Access

This article has been published open access under our Subscribe to Open programme, made possible through the support of our subscribing institutions, learn more here: https://portlandpress.com/pages/open_access_options_and_prices#conditional

## Abbreviations

CAT: cancer-associated thrombosis
CTCs: circulating tumor cells
CTMs: circulating tumor microemboli
DVT: deep vein thrombosis
VTE: venous thromboembolism
